# Effectiveness of a Mobile-Based Self-Regulation Training on Youths’ Affect

**DOI:** 10.3390/healthcare14010133

**Published:** 2026-01-05

**Authors:** Anouk Aleva, Annemiek Karreman, Loes H. C. Janssen, Anouk Vroegindeweij, Marcel A. G. van Aken, Christel J. Hessels, Odilia M. Laceulle

**Affiliations:** 1 HYPE Centre of Expertise on Early Intervention for Borderline Personality Disorder, GGz Centraal, 3812 GV Amersfoort, The Netherlands; 2 Department of Developmental Psychology, Utrecht University, 3584 CS Utrecht, The Netherlands; 3 Department of Medical and Clinical Psychology, Center of Research on Psychological Disorders and Somatic Diseases, Tilburg University, 5037 AB Tilburg, The Netherlands; 4 Department of Social Pediatrics, Wilhelmina Children’s Hospital, University Medical Center Utrecht, Utrecht University, 3584 EA Utrecht, The Netherlands

**Keywords:** self-regulation training, affect, youth, mobile-based, self-control, emotion regulation strategies, prevention

## Abstract

**Background:** The rising prevalence and enduring impact of mental health problems in youth have intensified the call for population-level prevention. Low positive and high negative affect in childhood are vulnerability factors for mental health problems in adolescence. Supporting youth in managing affect during early adolescence may foster mental health preventively. Self-regulation training has shown promise in this regard. Moreover, its parallels with Behavioral Activation (BA) align with the recommendation to adapt evidence-based clinical interventions into scalable, accessible formats for prevention. **Methods:** This study examined whether a 12-day mobile-based self-regulation training, consistent with BA principles and delivered in an innovative digital format, could increase positive and decrease negative affect in a sample of 156 youths (*M_age_* = 10.0). **Results:** No significant group differences emerged in affect change over time, and neither baseline levels of self-control nor emotion regulation strategies moderated the effects. **Conclusions:** The findings suggest that low-intensity mobile-based interventions may be insufficient to produce meaningful affect change in youth. The potential need to shift from universal prevention strategies to more selective approaches targeting at-risk youth is discussed.

## 1. Introduction

Youth mental health has been declining for over two decades [1]. As mental health problems often first emerge during adolescence, with most disorders having their onset around age 15 [2], they frequently disrupt psychosocial development and have long-term consequences [3,4]. The rising prevalence and enduring impact of mental health problems in youth have intensified the call for population-level prevention [5,6,7]. Preventive efforts targeting youth in early adolescence are considered particularly promising, given their potential to mitigate the emergence of later mental health problems [6]. Low positive affect and high negative affect in childhood have been identified as vulnerability factors for later mental health problems [8,9]. As such, supporting youth to effectively manage their affect might offer a promising target in light of prevention [10]. Self-regulation training has shown potential for increasing positive affect and reducing negative affect [11]. Moreover, its parallels with Behavioral Activation (BA) [12] align with the recommendation to adapt evidence-based clinical interventions into scalable, accessible formats for prevention [7,13]. However, training approaches have varied and the extent to which individual differences influence their impact remains unclear. Accordingly, the current study examined whether a 12-day mobile-based self-regulation training, which aligned with BA principles, improved affect in youth. Additionally, given the central role of self-control and emotion regulation to self-regulation [14], it was examined whether individual differences in these skills at baseline moderated the effectiveness of the training.

Self-regulation encompasses the process of flexibly managing behavior, attention, emotions, and cognitive strategies in response to internal and external cues, aimed at attaining personal goals [15]. Early in life, self-regulation predominantly involves controlling impulses [16,17]. During childhood, it develops into a more complex process [18,19]. Positive changes in self-regulation in early childhood—such as improved behavioral and emotional control—have been associated with a reduced risk of negative outcomes in adolescence, including mental health problems and school truancy [20]. As self-regulation continues to mature, youth become increasingly capable of independently applying its core components: setting goals, monitoring progress, and adjusting behavior [21,22]. Research suggests that strengthening self-regulation in youth can support both the maintenance and enhancement of positive affect throughout adolescence [23]. Moreover, a stronger sense of self-efficacy to regulate positive and negative affect has been found to be prospectively linked to better management of key adolescent developmental challenges, such as managing academic goals and resisting social pressures for antisocial activities [24].

Although self-regulation trainings have shown promising results in improving affect in youth [11,25,26], approaches have differed considerably in several key aspects. Studies have varied in the specific self-regulatory skills targeted, the extent of involvement of support figures (e.g., parents, teachers), and the focus of the training. For example, youth self-regulation trainings have focused on goal setting (e.g., planning for the future, reflecting on aspirations, monitoring progress), stress management (e.g., relaxation techniques, mindfulness), and cognitive restructuring (e.g., re-interpreting situations) [25,26]. Due to this substantial heterogeneity in training content, there is currently no consistent evidence linking a specific self-regulatory skill or approach to improvements in affect. Moreover, small sample sizes and the lack of a clear theoretical framework in some studies further complicate the evaluation and comparison of their effectiveness.

The (developmental) strengthening of self-regulatory processes has been suggested to enable individuals to pursue their goals, overcome challenges, build self-confidence, and engage in rewarding activities [27,28]. Effective self-regulation has been associated with higher levels of positive affect and well-being [29,30], whereas difficulties in self-regulation have been linked to elevated levels of negative affect [31]. Accordingly, strengthening self-regulation has been a core component of interventions targeting clinical levels of negative affect, such as depressive symptoms, particularly through Behavioral Activation (BA). BA aims to improve mood and daily functioning by increasing engagement in personally meaningful and rewarding activities, using techniques such as activity monitoring and scheduling [12]. Beyond its well-established effectiveness in treating clinical depression [32], BA has also been found to enhance well-being in non-clinical populations of adults [33], which algins with its conceptual overlap with self-regulation. Its relatively simple and structured approach has been suggested to make it particularly scalable and developmentally adaptable for youth [34]. In recent years, research on BA in youth has been growing, supporting its feasibility, acceptability, and its potential to improve mood [34,35,36]. During the COVID-19 pandemic, a single-session, online adaptation of BA was implemented as a public health intervention for youth and was shown to reduce depressive symptoms and hopelessness, while increasing agency [37]. However, rigorous studies are needed to establish whether BA’s broader benefits on affect observed in adults can be replicated in youth.

Although BA and self-regulation have emerged from different academic traditions—clinical psychology and developmental psychology, respectively—their core techniques and components show considerable overlap. Both emphasize elements such as personally meaningful goal-setting, progress (self-)monitoring, and goal-related behavioral adjustments or scheduling [12,21,22]. BA’s effectiveness in improving affect suggests that a self-regulation training targeting similar processes might yield comparable outcomes. Following recommendations to adapt evidence-based clinical interventions into scalable and accessible formats for population-level prevention in youth [7,13], aligning a self-regulation training with core techniques from BA may represent a promising approach to promote effective affect management in this population. To enhance the developmental fit and engagement with BA in youth, recommended adaptations include flexible delivery formats (e.g., face-to-face or online), tailored materials, and the creation of developmentally appropriate behavioral activity lists [34,36]. From a self-regulation perspective, a developmentally informed training should tailor to youth’s evolving cognitive and emotion capacities [38,39]. In early adolescence, youth begin to develop greater autonomy and intrinsic motivation in goal-setting, prioritization, and planning [15,40]. This suggest that a self-regulation training during this period might be particularly effective when it supports these emerging capacities.

Successful self-regulation requires flexible adaptation of thoughts, emotions, and behavior to immediate environmental cues in the pursuit of long-term, personally meaningful goals [41]. Failures in self-regulation have been found to be triggered by difficulties in resisting temptation or managing emotional distress [42]. The ability to control behavioral and emotional impulses—defined as self-control and emotion regulation, respectively—might therefore serve as basic skills necessary to benefit from training more complex self-regulatory processes. Both self-control and emotion regulation have been associated with more successful self-regulation [43,44]. Individual differences in these basic self-regulatory skills might influence who is most likely to benefit from more complex regulatory training. Greater self-control and more frequent use of adaptive emotion regulation skills have been suggested to free up cognitive resources, thereby facilitating the implementation of more complex self-regulation processes [45,46]. As such, youth with greater self-control and more frequent use of adaptive emotion regulation strategies might benefit more from a self-regulation training.

Given the increasingly digital lifestyles of youth, a mobile-based self-regulation training holds promise for promoting active engagement. Through daily app-prompts, such a training may scaffold long-term goal pursuit by supporting progress monitoring and scheduling sub-goals. Evidence from the field of BA also indicates that delivering BA through mobile apps is feasible and shows promising outcomes [47]. Moreover, app-prompts may provide a balance between autonomy and external support for youth. Mobile delivery also facilitates the integration of practice into daily life [48], thereby enhancing personal relevance and promoting regular engagement—both of which have been associated with increases in overall self-regulatory capacity [39,49]. To date, however, mobile-based approaches have not been systematically applied and evaluated in the context of self-regulation training and affect in youth [11,26].

### Aims of This Study

The current study examined whether a 12-day mobile-based self-regulation training could improve affect in youth. The training aligned with Behavioral Activation (BA) principles, which is an evidence-based clinical intervention for improving mood and functioning. To our knowledge, no previous study has combined self-regulation training and Behavioral Activation (BA) in a preventive, scalable, app-based training for youth. Youth were recruited from Dutch primary schools and randomly assigned to a training or control group. The training group set a goal for the 12-day period and received daily app-prompts supporting progress monitoring and the planning of goal-related behaviors. It was hypothesized that youth in this group would exhibit increases in positive affect and decreases in negative affect over the course of the 12-day period. Baseline levels of self-control and adaptive emotion regulation strategies were examined as potential moderators, with individuals with higher initial levels being expected to show greater improvements in affect. The findings can shed light on the potential of adapting evidence-based clinical interventions into brief, population-level prevention formats for youth.

## 2. Method

### 2.1. Sample

**Figure 1:**
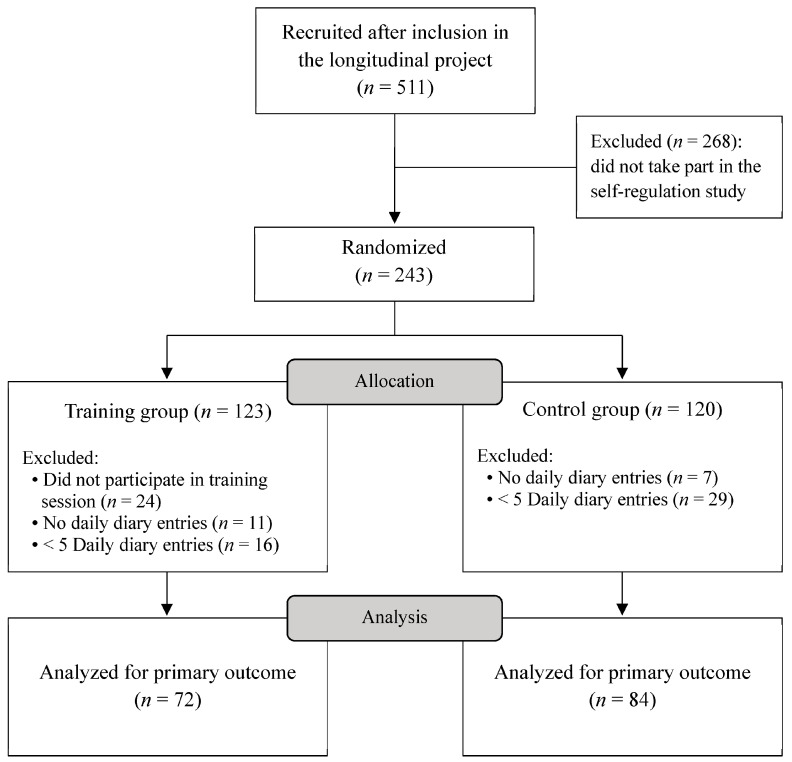
Participant Flow Diagram.

The current study was part of a larger longitudinal project on vulnerability and resilience in youth that started in 2017 [50]. Youth aged 9 to 11 were recruited from 24 Dutch primary schools. The self-regulation training study was conducted during the first wave of the longitudinal project. Figure 1 presents the participant flow diagram. Participants who chose to join the current study did not differ from those who did not in sex distribution (χ^2^(1, *N* = 511) = 1.31, *p* = 0.25) or the use of adaptive emotion regulation strategies (*t*(486) = −0.47, *p* = 0.64). Comparisons were not made for age, as all participants were recruited in the same school year, or for self-control, which was only assessed in those included in the self-regulation study.

The power analysis for the longitudinal project indicated that 500 participants were required to examine the key risk and protective factors for resilience [50]. No formal a priori power analysis was conducted for the current study, as procedures for multilevel models with daily diary data were still under development at the time. However, the methodological literature suggested that models with at least 50 participants provide unbiased estimates [51], which served as a guideline for both groups in the current study.

Initially, 243 participants were included and randomized in the current study. Participants who did not attend the training session or failed to complete any daily diary entries were excluded. Subsequently, a compliance threshold of ≥5 out of 12 diary entries was applied. This threshold was based on three considerations related to psychometric and training validity. First, prior research has recommended that daily diary analyses should include participants with mostly completed entries to reduce bias (e.g., ≥4 out of 7 days) [44]. Second, to enable a reliable estimation of individual variability through random effects, a minimum of five completed entries is advised [45]. Third, when a daily diary study includes an intervention component, studies in adults have shown a minimum level of adherence to be important as non-compliance might significantly reduce potential intervention effects [46]. A total of 156 participants (78%) met the compliance threshold and were included in the analyses. No significant differences were found between compliant and non-compliant participants on the study variables (see Supplementary Materials Tables S1 and S2). Descriptive statistics of the sample are presented in Table 1.

### 2.2. Ethics, Consent and Data Sharing

The longitudinal project [50], including the current study, was approved by the Tilburg School of Social and Behavioral Sciences Ethics Review Board (EC-2016.63). Prior to inclusion, informed consent was obtained from youth and their primary caretaker. The current study was not preregistered. Analysis scripts are openly available on the Open Science Platform (https://osf.io/u7e2w). In line with privacy regulations in place at the start of the project, the data are available upon reasonable request from the authors.

### 2.3. Procedure

Questionnaire data for the longitudinal project were collected from youth in their classroom by trained research assistants. Youth were then invited to participate in the current study on the self-regulation training, which began immediately thereafter. Those who chose to participate were randomly assigned to the training or control group using an allocation sequence generated in SPSS. The groups received further instructions separately. All participants downloaded the Ilumivu app onto a mobile phone—either their own, a parent’s, or one provided by the study. The app sent a daily prompt at 06.30 PM for 12 consecutive days and included brief questions about participants’ emotional experiences and a general impression of their day (i.e., daily entry). Each daily entry took <2 min to complete. Participants completed a practice entry during inclusion. Research assistants were available throughout the study to provide support in case of technical issues.

To encourage compliance, a tablet was raffled among participants who completed ≥ 10 entries. Additionally, entry completion was monitored and a research assistant provided reminders when necessary. At each prompt, the control group solved a simple math problem, while the training group completed the self-regulation training.

### 2.4. Self-Regulation Training

The self-regulation training comprised two components: (1) setting a goal for the 12-day period, and (2) daily monitoring and planning goal-related behaviors. During inclusion, participants received a list of 10 goals and were asked to choose one that they felt was relevant for them to work on. Research assistants provided a brief explanation of the goals using a structured script and were available to answer questions and provide clarification if necessary. The list of goals covered four domains—externalizing behavior, internalizing behavior, lifestyle, and responsibility—with 15%, 35%, 25%, and 25% of participants selecting goals in these domains, respectively (further details in Supplementary Material Table S3). After entering their chosen goal in the app, participants received a card listing adaptive goal-related behaviors (e.g., for the goal “less fighting” an alternative behavior was “If I notice that something is making me angry, I will tell someone”). At each daily prompt, participants selected a goal-related behavior from the card to practice the following day. The next day, they reflected on their progress in the app by indicating whether they completed their chosen goal-directed behavior, were satisfied with their efforts, felt responsible for the outcome, and—if they did not practice—explained why. Participants then selected a new behavior or could choose to repeat the same one if they felt it had not yet worked out. Completing the daily self-regulation training component took approximately five minutes.

### 2.5. Measures

#### 2.5.1. Positive and Negative Affect

Positive and negative affect were measured through the daily diary questions (i.e., daily entries). Participants indicated the extent to which they experienced emotions on a 5-point Likert scale (ranging from 1 = *not at all* to 5 = *very much* with accompanying smiley faces). The emotions were based on the Positive and Negative Affect Schedule (PANAS) [52,53]. Positive affect was calculated as the mean score of relaxed, satisfied, confident, happy, energetic, and excited. Negative affect was calculated from the items unhappy, disappointed, angry, nervous, and irritated. Between-person correlations ranged from moderate to very strong for both positive (*r* = 0.47–0.88, *p* < 0.001) and negative emotions (*r* = 0.50–0.91, *p* < 0.001). Within-person correlations ranged from small to moderate-to-strong for positive emotions (*r* = 0.13–0.66, *p* < 0.001), and from small to strong for negative emotions (*r* = 0.08–0.72, *p* < 0.001) (see Supplementary Material Tables S4 and S5 for details).

#### 2.5.2. Self-Control

The Brief Self Control Scale (BSCS) [54] was administered at baseline as a measure of self-control. The wording of this 13-item self-report questionnaire was slightly adapted for the current study to make the items more age-appropriate. For example, “I am able to work effectively toward long-term goals” was changed to “I can work well on goals that I want to achieve later”. The content validity of the scale was considered to be retained as the adaptations were only minor. Participants rated each item on a 5-point Likert scale (ranging from 1 = *not at all like me* to 5 = *very much like me*). A mean score was calculated, with a higher score reflecting greater self-control. Cronbach’s alpha in the current study was 0.80.

#### 2.5.3. Adaptive Emotion Regulation Strategies

The short version of the Cognitive Emotion Regulation Questionnaire (CERQ-short) [55] was administered at baseline to assess the use of adaptive emotion regulation strategies. The CERQ-short is an 18-item self-report questionnaire. Participants were asked to indicate how often they typically use a specified cognitive strategy in response to negative life events. Responses were given on a 5-point Likert scale (1 = *(almost) never* to 5 = *(almost) always*). In the current study, the overall mean of the 10 items reflecting adaptive emotion regulation strategies—acceptance, positive refocusing, refocus on planning, positive reappraisal, and putting into perspective—was used [56]. A higher score indicated greater use of adaptive emotion regulation strategies. Cronbach’s alpha in the current study was 0.78.

### 2.6. Statistical Analyses

Data preparation was undertaken in IBM SPSS Statistics (v29) and analyses were performed in R version 4.4.0 [57]. Linear mixed-effects modeling—also referred to as multilevel modeling—was conducted using the lme4 package [58]. A series of models was run in a stepwise fashion, separately for positive and negative affect, with the control group set as the reference group. Results were considered significant at *p* < 0.05.

First, a model predicting affect with *day* (time), *group* and their interaction as fixed effects was fitted to test whether there was an average trend in affect across days for each group. A random intercept for each participant was included to account for individual differences in affect at baseline. Next, the model was extended to include a random slope for *day* (time), allowing participants to vary in their rate of change in affect over time. A likelihood ratio test was used to compare the fit of the models. The best-fitting model was then expanded with two three-way interaction terms: one including self-control and another including adaptive emotion regulation strategies. Because of missing questionnaire data, these models were run on a reduced sample (*n* = 136 for self-control; *n* = 144 for adaptive emotion regulation strategies). Following multilevel modeling guidelines, these predictors were grand-mean centered [59].

Finally, post hoc exploratory analyses were conducted to examine whether additional factors might have moderated the effects in order to better understand the observed results. Sex, baseline positive affect, and baseline negative affect were first explored. The best-fitting model was then expanded to include a three-way interaction term separately for each of these variables (with baseline affect first grand-mean centered). Next, the potential moderating role of task completion (grand-mean centered) was also examined within the training group.

## 3. Results

### 3.1. Group Differences in Change in Positive and Negative Affect

Descriptive statistics are presented in Table 1. Comparisons of baseline affect between groups showed no significant differences in either positive (*t*(142) = 0.25, *p* = 0.80) or negative affect (*t*(149) = −0.92, *p* = 0.36), indicating that the groups were comparable at the start of the study.

Table 2 presents the results of the model-building steps. The interaction between day and group was not significant for either positive or negative affect, indicating that changes in affect over time did not differ between the training and control group.

Although the model including random slopes for day provided a significantly better fit for both positive (χ^2^ (2) = 22.00, *p* < 0.001) and negative affect (χ^2^ (2) = 11.81, *p* = 0.003), the magnitude of the individual variation was small (see Supplementary Materials Figures S1 and S2).

### 3.2. Self-Control and Adaptive Emotion Regulation Strategies

When self-control and adaptive emotion regulation strategies at baseline were added to the models, neither significantly moderated the association between day and group for positive or negative affect.

As a secondary finding, both self-control and adaptive emotion regulation strategies emerged as significant main effects for positive affect. This indicates that participants with a higher level of self-control or a higher level of adaptive emotion regulation strategies at baseline reported higher levels of positive affect across the 12-day period.

### 3.3. Post Hoc Analyses

To gain further insight into the observed null findings, additional factors were explored as potential moderators. A brief summary is provided below, with detailed results reported in the Supplementary Materials (Section S1 Post Hoc Exploratory Results).

First, sex was examined. For both positive and negative affect, sex did not moderate changes in affect over time or differences between groups. Next, baseline affect was examined. Baseline positive and negative affect both moderated changes over time (positive affect; *B* = −0.03, *SE* = 0.04, *p* = 0.003; negative affect; *B* = 0.78, *SE* = 0.017, *p* < 0.001), such that higher baseline affect was associated with smaller changes in the corresponding affect over time. Baseline affect did not significantly moderate group effects on positive or negative affect. At the same time, positive and negative baseline affect did show main effects on their respective affect outcomes (positive affect: *B* = 0.81, *SE* = 0.06, *p* < 0.001; negative affect: *B* = 0.78, *SE* = 0.017, *p* < 0.001), indicating that higher baseline affect was associated with a higher level of the respective affect across the study period.

Finally, within the training group, task completion was explored as a potential moderator. Task completion was defined as the number of days that participants reported that they completed their chosen goal-directed behavior. Task completion did not moderate changes in positive or negative affect over time. However, a small main effect of task completion on negative affect did appear in this model (*B* = 0.05, *SE* = 0.02, *p* = 0.032).

Overall, these results should be interpreted with caution given the multiple post hoc tests and small sample size of the training group (*n* = 72).

## 4. Discussion

The current study examined whether a 12-day mobile-based self-regulation training could improve affect in youth by comparing a training and a control group. No differences were observed in changes in positive and negative affect over time between the groups, suggesting the training did not impact affect. Baseline levels of self-control and the use of adaptive emotion regulation strategies did not influence this outcome. However, the level of self-control and adaptive emotion regulation strategies at baseline both appeared positively associated with positive affect throughout the 12-day period, which is in line with previous research [60,61].

The development and testing of the self-regulation training in the current study was undertaken in response to calls for preventive approaches to youth mental health problems [5,6,7]. Its conceptual parallel to Behavioral Activation (BA) was motivated by recommendations to adapt evidence-based clinical interventions into scalable and accessible formats for population-level prevention [7,13]. The null findings regarding improvements in affect suggests that adapting clinically validated, evidence-based interventions into brief, population-level prevention formats—while preserving their active components—may be more challenging than anticipated.

Brief, online applications of BA have shown promise in improving outcomes in youth, such as reducing depressive symptoms, even with as little as a single session [37]. However, the null findings from the current study call into question on the universal applicability of this approach and suggest that perhaps a more intensive or multifaceted BA-aligned self-regulation training may be necessary to achieve changes in affect. Studies on self-regulation trainings in adults have demonstrated that a higher frequency of prompts can enhance self-regulation and, in turn, positively influence affect [62,63]. For example, a recent study showed that delivering three prompts within a 30 min session—targeting self-regulation processes such as goal management and behavioral adjustment—led to increases in positive affect [63]. These findings suggest that more frequent engagement through app-prompts might better scaffold self-regulation and support sustained effort, ultimately facilitating changes in affect. Such an increase in prompt frequency may need to be substantial, as post hoc analyses in the current study did not reveal a dose–response effect in the current study (i.e., task completion did not moderate changes in affect in the training group). However, there was no formal measure of the quality of youths’ engagement with the training included, leaving open the possibility that participants did not actively engage with the prompts in the current study.

Regarding the design of a self-regulation training for youth, incorporating additional training components may be necessary. Prior research suggests that elements such as problem-solving skills and caregiver involvement may be necessary to effectively enhance self-regulation and improve affect in youth [36,39]. Whereas a self-regulation training for children is typically delivered with, or even through, a supportive figure—often a caregiver or teacher [64]—adult BA-based interventions are focused on the individual [33]. Youth, however, occupy a distinct developmental period in between: their autonomy is growing, yet they may still benefit from guided support. In the current study, parents were informed through the information letter, and the first goal-directed behavior listed on each card was “I am going to tell my parents about my goal so they can help me a little”. However, parents were not formally involved in the training, leaving the initiative for seeking support with the youth. It is possible that more active involvement of parents or other supportive figures is needed to achieve change, while simultaneously keeping in mind youths’ growing need for autonomy.

A sample-related consideration when interpreting the lack of change in affect are the levels of positive and negative affect at the start of the study. Baseline scores for both positive and negative affect were already near the scale’s extremes (see Table 1 and the density plots in Figures S3 and S4 in the Supplementary Material), suggesting limited room for improvement over the 12-day period. This points to a potential ceiling effect for positive affect and floor effect for negative affect. Post hoc analyses further indicated that higher baseline positive and negative affect were associated with higher levels of the respective affect throughout the study and with smaller changes over time in the respective affect. Together, these patterns suggest that baseline levels may have constrained the potential for change during the study.

Nevertheless, in the context of preventive, population-level interventions, even small effects can be valuable, as they may translate into meaningful public health gains when implemented at a larger scale [65]. Moreover, previous research highlights the importance of moving beyond merely identifying statistical significance in such contexts and considering whether observed changes are meaningful from the participant’s perspective [66]. In adult population samples, a group-level change of ≥0.3 points on 5-point Likert scales of daily affect—similar to the current study—has been found to represent a noticeable shift, such as feeling “*a little more positive*” or “*a little less negative*” [66]. By this standard, meaningful improvements would have been possible in the current sample despite the baseline levels of affect. The absence of such change therefore suggests that the lack of effects was not solely due to floor or ceiling constraints, but likely reflects the limited effectiveness of the training.

Moving forward, research might focus less on increasing the average magnitude of population-level interventions and more on identifying subsets of best responders. For example, the online, single session BA intervention—which proved effective, though with small effect sizes—targeted youth from the general population but included only those meeting a threshold level of depressive symptoms [37]. Rather than applying universal prevention strategies that address risk factors across all youth, without distinguishing those at elevated risk, the field could shift toward selective prevention, targeting groups identified as sharing a significant risk factor [67]. For example, selective prevention programs for youth with parents with a mood or anxiety disorder have demonstrated significant reductions in the risk of developing depressive or anxiety symptoms in youth themselves [68]. A subsequent step would be indicated prevention, which targets individuals showing significant mental health problems but who do not yet meet diagnostic criteria for a disorder. Online indicated preventive interventions for youth with emerging mental health problems have shown promise, including improvements in well-being and resilience [69].

While considerable thought went into the design of the current study and the data were thoroughly examined to provide a balanced answer to the research questions, several limitations should be acknowledged. First, the timing of daily diary entries may have influenced the results. The daily prompt time was predetermined by the research team to align with the typical Dutch family schedule (i.e., occurring shortly after the common diner time), based on the assumption that this would maximize completion and facilitate parental support if necessary. Nevertheless, variations in individual family schedules may have affected compliance. As the exact timing of diary entries could not be retrieved, the impact of potential variation could not be examined. Second, although the math problems in the control group were designed to match youths’ respective school year, no measures of perceived difficulty or engagement were included. As a result, it remains unclear how the motivational and cognitive load of these tasks compared to that of the self-regulation training. Third, the potential impact of the type of goal that was selected was not examined in the current study in order to avoid overstraining the model given the available data. However, the influence of goal domain may be a relevant avenue for future research. Fourth, although self-control and the use of adaptive emotion regulation were theoretically motivated potential moderators, we did not interpret their lack of moderation in the current study. This decision was based on the absence of overall training effects and the floor and ceiling considerations in our sample. Further research is needed to determine whether these skills play a substantial role in influencing self-regulation training effects in other contexts (e.g., in at-risk samples). Finally, future research might consider incorporating more frequent assessments of affect throughout the day, as these could better capture emotional fluctuations, which may last from a few seconds to several hours [70]. Early adolescence is particularly characterized by emotional instability [71], suggesting that a more nuanced understanding of affect could be especially important in this group.

## 5. Conclusions

The current study responded to the call for preventive approaches to the rising prevalence of youth mental health problems. An innovative mobile-based self-regulation training, drawing on principles of Behavioral Activation (BA), was designed to improve affect in youth. The null findings suggest that this low-intensity training may have been too minimal to meaningfully influence affect. While further research is needed before drawing strong conclusions, these results represent an important step toward a more critical and nuanced perspective on the potential of digital interventions for youth mental health prevention. By offering a robust test of a basic, standalone intervention, this study indicates that improving affect in youth is unlikely to be achieved through simple or low-effort solutions. Meaningful change may require more intensive training and additional components, each of which should be systematically evaluated for its contribution to effectiveness. It may also be more effective to shift from universal population-level preventive strategies to selective prevention, targeting youth with identifiable risk factors. Ultimately, refining digital interventions and thoroughly evaluating their effectiveness will help advance the field toward scalable options that foster resilience and mental health during adolescence.

## Figures and Tables

**Table 1:** Descriptive Statistics for the Total Sample, Training Group, and Control Group.

	Total Sample	Training Group	Control Group
	*N* = 156	*n* = 72	*n* = 84
Age, *M* (*SD*)	10.0 (0.4) ^a^	9.96 (0.4) ^b^	10.1 (0.4) ^c^
Girl/Boy	82/74	41/31	41/43
Self-Control, *M* (*SD*)	3.8 (0.7) ^d^	3.8 (0.7) ^e^	3.8 (0.7) ^f^
Adaptive Emotion Regulation Strategies, *M* (*SD*)	2.8 (0.7) ^g^	2.8 (0.6) ^h^	2.8 (0.7) ^i^
Positive Affect at Day 1, *M* (*SD*)	4.2 (0.6)	4.2 (0.6)	4.2 (0.5)
Positive Affect at Day 12, *M* (*SD*)	4.3 (0.6)	4.4 (0.6)	4.2 (0.6)
Negative Affect at Day 1, *M* (*SD*)	1.5 (0.5)	1.5 (0.5)	1.6 (0.5)
Negative Affect at Day 12, *M* (*SD*)	1.5 (0.6)	1.5 (0.7)	1.5 (0.6)

^a^ *n* = 147; ^b^ *n* = 67; ^c^ *n* = 80; ^d^ *n* = 136; ^e^ *n* = 71; ^f^ *n* = 65; ^g^ *n* = 144; ^h^ *n* = 67; ^i^ *n* = 77.

**Table 2:** Stepwise Models Predicting Positive and Negative Affect Including Day, Group, Self-Control, and Adaptive Emotion Regulation Strategies.

		B	SE	*p*-Value	95% CI
**Positive Affect**					
**Step 1**	Day	0.01	0.01	0.083	−0.00, 0.02
	Group	0.01	0.09	0.904	−0.16, 0.18
	Day × Group	0	0.01	0.865	−0.02, 0.01
**Step 2**	Day	0.01	0.01	0.079	−0.00, 0.02
	Group	0.13	0.08	0.127	−0.04, 0.29
	Day × Group	−0.01	0.01	0.205	−0.03, 0.01
	Self-Control	0.18	0.06	0.003 **	0.06, 0.30
	Adaptive Emotion Regulation Strategies	0.14	0.06	0.016 *	0.03, 0.25
**Step 3**	Day	0.01	0.01	0.060	−0.00, 0.02
	Group	0.11	0.09	0.210	−0.06, 0.28
	Day × Group	−0.01	0.01	0.284	−0.02, 0.01
	Self-Control	0.24	0.09	0.006 **	0.07, 0.42
	Day × Self-Control	0	0.01	0.623	−0.02, 0.01
	Group × Self-Control	−0.02	0.13	0.903	−0.27, 0.24
	Day × Group × Self-Control	0.02	0.01	0.175	−0.01, 0.04
**Step 4**	Day	0.01	0.01	0.106	−0.00, 0.02
	Group	0.05	0.08	0.569	−0.12, 0.21
	Day × Group	0	0.01	0.707	−0.02, 0.01
	Adaptive Emotion Regulation Strategies	0.26	0.10	0.009 **	0.07, 0.45
	Day × Adaptive Emotion Regulation Strategies	0	0.01	0.696	−0.01, 0.02
	Group × Adaptive Emotion Regulation Strategies	−0.1	0.12	0.431	−0.34, 0.14
	Day × Group × Adaptive Emotion Regulation Strategies	−0.01	0.01	0.60	−0.03, 0.02
**Negative Affect**					
**Step 1**	Day	0	0.01	0.663	−0.01, 0.01
	Group	0.01	0.07	0.903	−0.13, 0.15
	Day × Group	0	0.01	0.590	−0.02, 0.01
**Step 2**	Day	0	0.01	0.381	−0.02, 0.01
	Group	−0.06	0.08	0.407	−0.21, 0.09
	Day × Group	0.01	0.01	0.407	−0.01, 0.02
	Self-Control	−0.09	0.05	0.069	−0.20, 0.01
	Adaptive Emotion Regulation Strategies	−0.03	0.05	0.533	−0.13, 0.07
**Step 3**	Day	0	0.01	0.788	−0.01, 0.01
	Group	−0.05	0.07	0.512	−0.19, 0.10
	Day × Group	0	0.01	0.793	−0.01, 0.02
	Self-Control	−0.03	0.08	0.731	−0.17, 0.12
	Day × Self-Control	−0.01	0.01	0.177	−0.03, 0.00
	Group × Self-Control	−0.09	0.11	0.419	−0.31, 0.13
	Day × Group × Self-Control	0	0.01	0.930	−0.02, 0.02
**Step 4**	Day	−0.01	0.01	0.344	−0.02, 0.01
	Group	−0.01	0.07	0.890	−0.15, 0.13
	Day × Group	0	0.01	0.958	−0.01, 0.01
	Adaptive Emotion Regulation Strategies	−0.15	0.09	0.081	−0.32, 0.02
	Day × Adaptive Emotion Regulation Strategies	0	0.01	0.938	−0.02, 0.02
	Group × Adaptive Emotion Regulation Strategies	0.18	0.11	0.093	−0.03, 0.40
	Day × Group × Adaptive Emotion Regulation Strategies	0	0.01	0.979	−0.02, 0.02

* *p* < 0.05. ** *p* < 0.01. *Note*. All models include a random intercept and a random slope for *day* (time) and were estimated separately for positive and negative affect. Step 1 includes the change in affect over time and by group, as well as their interaction. In subsequent steps, the a priori determined moderators were added. Step 2 includes self-control and the use of adaptive emotion regulation strategies separately and without interaction terms. Step 3 includes self-control and its interaction with time and group. Step 4 includes adaptive emotion regulation strategies and its interaction with time and group. Because of missing questionnaire data, the models including self-control and adaptive emotion regulation strategies were based on a reduced sample (*n* = 136 for self-control; *n* = 144 for adaptive emotion regulation strategies).

## Data Availability

The data presented in this study are available on request from the corresponding author. The data are not publicly available due to privacy restrictions.

## Supplementary Materials

The following supporting information can be downloaded at: https://www.mdpi.com/article/10.3390/healthcare14010133/s1, Figure S1: Range of Unstandardized Individual Slopes of Day (Time) to Positive Affect; Figure S2: Range of Unstandardized Individual Slopes of Day (Time) to Negative Affect; Figure S3: Density Plot of Positive Affect at Baseline (*N* = 156); Figure S4: Density Plot of Negative Affect at Baseline (*N* = 156); Table S1: Descriptives of Total Sample, Compliant, and Non-Compliant Participants; Table S2: Goal Selection in Training Group (Total, Compliant, and Non-Compliant Participants); Table S3: Domains and Goals in the Training Group; Table S4: Correlations Positive Affect; Table S5: Correlations Negative Affect; Section S1: Post Hoc Exploratory Results.
