# Supplementary material for: Effectiveness of a Mobile-Based Self-Regulation Training on Youths’ Affect
Source: Healthcare (Basel). 2026 Jan 5;14(1):133. doi: 10.3390/healthcare14010133 (PMC12785396; doi:10.3390/healthcare14010133)
Supplement: Supplementary file 1 [file healthcare-14-00133-s001.zip › healthcare-4004329-supplementary.pdf]

Supplementary Material

Comparison of Compliant and Non-Compliant Participants	**2**
Goals in Training Group	**4**
Correlations Emotions Comprising Positive and Negative Affect	**5**
Distribution of Unstandardized Individual Slopes	**6**
Visualization of Distribution of Baseline Positive and Negative Affect	**8**
Post Hoc Exploratory Results	**10**

Comparison of Compliant and Non-Compliant Participants

Table S1. Descriptives of Total Sample, Compliant, and Non-Compliant Participants

	**Total Sample**	**Compliant (≥ 5 Completed Days)**	**Non-Compliant (< 5 Completed Days)**	**Test Statistic**
	N = 201*	n = 156	n = 45	
Age, *M* (*SD*)	10.03 (0.41)[a]	10.02 (0.41)[b]	10.08 (0.42)[c]	$t(58) = 0.85$, $p = .397$
Girl / Boy	104 / 97	82 / 74	22 / 23	X^2 (1, N = 201) = 0.07, $p = .791$
Training / Control	88 / 113	72 / 84	16 / 29	X^2 (1, N = 201) = 1.19, $p = .275$
Completed Daily Diary Assessments, *Mdn* (range)	8 (1 – 12)	9 (5 – 12)	3 (1 – 4)	NA
Self-Control, *M* (*SD*)	3.80 (0.66)[d]	3.78 (0.67)[e]	3.86 (0.65)[f]	$t(56) = 0.67$, $p = .508$
Adaptive Emotion Regulation Strategies, *M* (*SD*)	2.80 (0.69)[g]	2.80 (0.69)[h]	2.78 (0.66)[i]	$t(63) = -0.18$, $p = .861$
Positive Affect, *M* (*SD*)**	4.29 (0.60)	4.29 (0.61)	4.32 (0.49)	$t(149) = 0.74$, $p = .463$
Negative Affect, *M* (*SD*)**	1.49 (0.57)	1.49 (0.56)	1.54 (0.65)	$t(129) = 0.83$, $p = .410$

[a] n = 186; [b] n = 147; [c] n = 39; [d] n = 172; [e] n = 136; [f] n = 36; [g] n = 183; [h] n = 144; [i] n = 39

* Of the 243 participants that were randomized (as depicted in Figure 1 in the manuscript), 18 did not provide any diary entries and were therefore excluded. In addition, 24 participants from the training group did not attend the first assessment. They were also excluded as they missed essential training instructions.

** In the compliant subsample, 1872 entries were expected per affect, with 468 missing for positive affect and 467 for negative affect. In the non-compliant subsample, 540 assessments were expected per affect, with 423 missing for positive affect and 424 for negative affect.

Table S2. Goal Selection in Training Group (Total, Compliant, and Non-Compliant Participants)

	Total Sample	Compliant (≥5 Completed Days)	Non-Compliant (≤5 Completed Days)
**Goals**	$N = 88$	$n = 72$	$n = 16$
Externalizing<br>- Become less angry and fight less<br>- Be nicer and stop teasing or bullying*	12	11	1
Internalizing<br>- Stand up for myself<br>- Complain or whine less when things do not go my way<br>- Worry less	30	25	5
Lifestyle<br>- Watch less television, game less, or reduce time spent on my phone<br>- Eat less candy<br>- Go to bed on time	24	18	6
Responsibility<br>- Pay more attention and listen better in class or at home<br>- Keep up with my homework, help more often with household chores or with the pet	22	18	4

* None of the participants selected this goal.

Goal selection did not significantly differ between the compliant and non-compliant participants, X^2 (8, $N = 88$) = 3.12, $p = 0.926$

Goals in Training Group

Table S3. Domains and Goals in the Training Group

Domain	Goals	Percentage of Participants Choosing Domain
Externalizing behavior	- Become less angry and less fighting<br>- Be nicer and stop teasing or bullying*	15.3 %
Internalizing behavior	- Stand up for myself<br>- Complain or whine less when things do not go my way<br>- Worry less	34.7 %
Lifestyle	- Watch less television, game less, or reduce time spent on my phone<br>-Eat less candy<br>- Go to bed on time	25 %
Responsibility	- Pay more attention and listen better in class or at home<br>- Keep up with my homework, help more often with household chores or with the pet	25 %

Note. 14 participants did not choose a goal.

* None of the participants chose this goal.

Correlations Emotions Comprising Positive and Negative Affect

Table S4. Correlations Positive Affect

**Variable**	**1.**	**2.**	**3.**	**4.**	**5.**	**6.**	**7.**
1. Relaxed	-	0.31**	0.27**	0.30**	0.20**	0.24**	0.63**
2. Satisfied	0.74**	-	0.27**	0.40**	0.28**	0.31**	0.66**
3. Confident	0.61**	0.63**	-	0.23**	0.13**	0.19**	0.55**
4. Happy	0.66**	0.72**	0.47**	-	0.28**	0.34**	0.66**
5. Energetic	0.58**	0.53**	0.47**	0.56**	-	0.31**	0.59**
6. Excited	0.74**	0.68**	0.57**	0.64**	0.68**	-	0.66**
7. Positive Affect Combined	0.88**	0.86**	0.77**	0.79**	0.77**	0.88**	-

Note. Between-person correlations are presented under the diagonal, within-person correlations are presented above the diagonal.

** $p < 0.001$

Table S5. Correlations Negative Affect

**Variable**	**1.**	**2.**	**3.**	**4.**	**5.**	**6.**	**7.**
1. Sad	-	0.46**	0.42**	0.34**	0.09**	0.29**	0.68**
2. Unhappy	0.83**	-	0.46**	0.33**	0.10**	0.31**	0.66**
3. Disappointed	0.70**	0.79**	-	0.40**	0.17**	0.37**	0.72**
4. Angry	0.73**	0.77**	0.81**	-	0.10**	0.43**	0.67**
5. Nervous	0.50**	0.57**	0.65**	0.59**	-	0.08**	0.44*
6. Irritated	0.58**	0.65**	0.72**	0.75**	0.58**	-	0.65**
7. Negative Affect Combined	0.83**	0.89**	0.91**	0.90**	0.77**	0.84**	-

Note. Between-person correlations are presented under the diagonal, within-person correlations are presented above the diagonal.

* $p < 0.01$, ** $p < 0.001$

Distribution of Unstandardized Individual Slopes

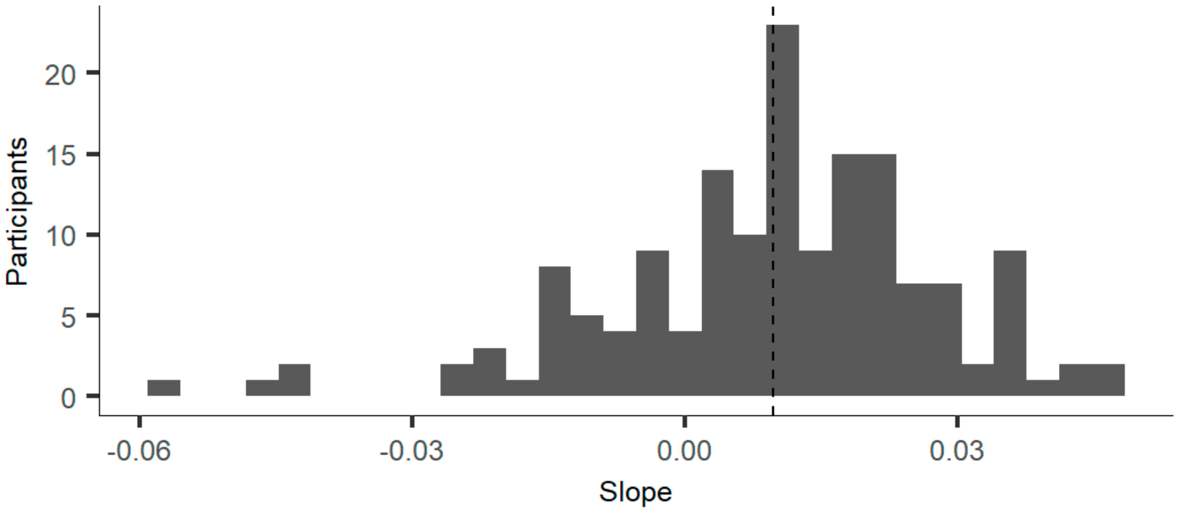

Figure S1. Range of Unstandardized Individual Slopes of Day (Time) to Positive Affect.

The vertical dashed line represents the mean slope across participants, which was 0.0097. This indicates that the average slope for day (time) to positive affect was positive. The standard deviation ranges from -0.0555 to 0.0483 (i.e., random variation in slopes). This range covers both negative (decrease) and positive (increase) values. Because the values are small relative to the 5-point Likert scale on which positive affect was measured, both the average slope as well as individual variation herein can be considered small in magnitude.

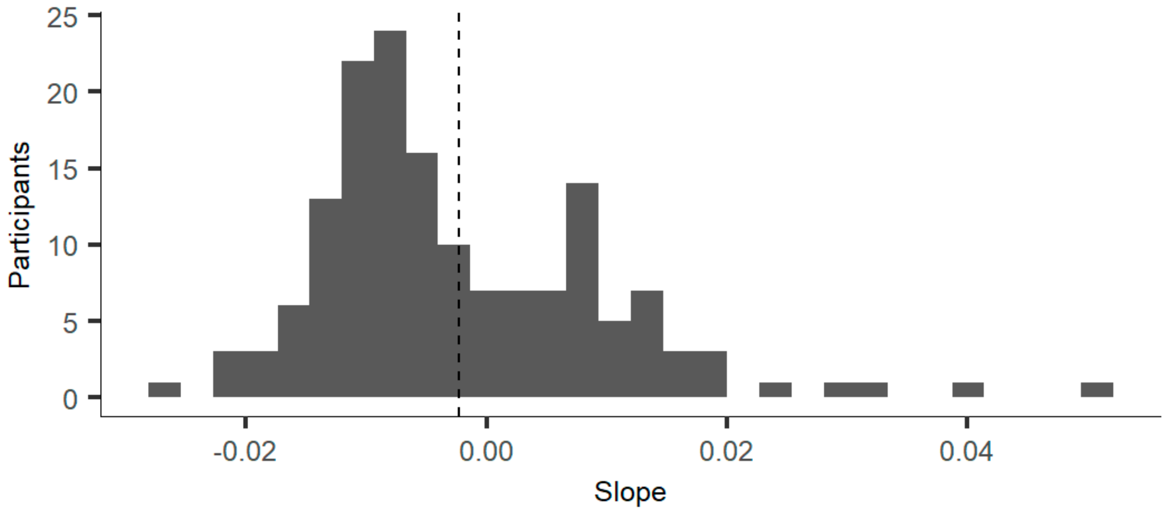

Figure S2. Range of Unstandardized Individual Slopes of Day (Time) to Negative Affect.

The vertical dashed line represents the mean slope across participants, which was -0.002. This indicates that the average slope for time (*day*) to negative affect was negative. The standard deviation ranges from -0.0263 to 0.0513 (i.e., random variation in slopes). This range covers both negative (decrease) and positive (increase) values. Because the values are small relative to the 5-point Likert scale on which negative affect was measured, both the average slope as well as individual variation herein can be considered small in magnitude.

Visualization of Distribution of Positive and Negative Affect

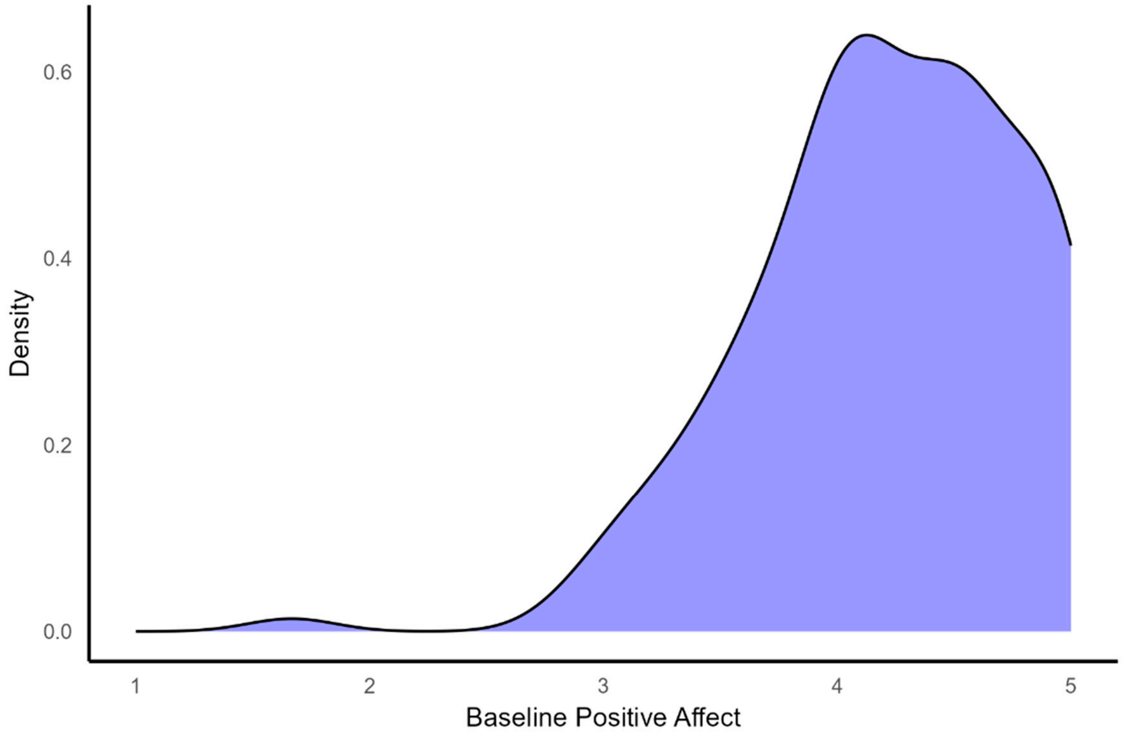

Figure S3. Density Plot of Positive Affect at Baseline (*N* = 156)

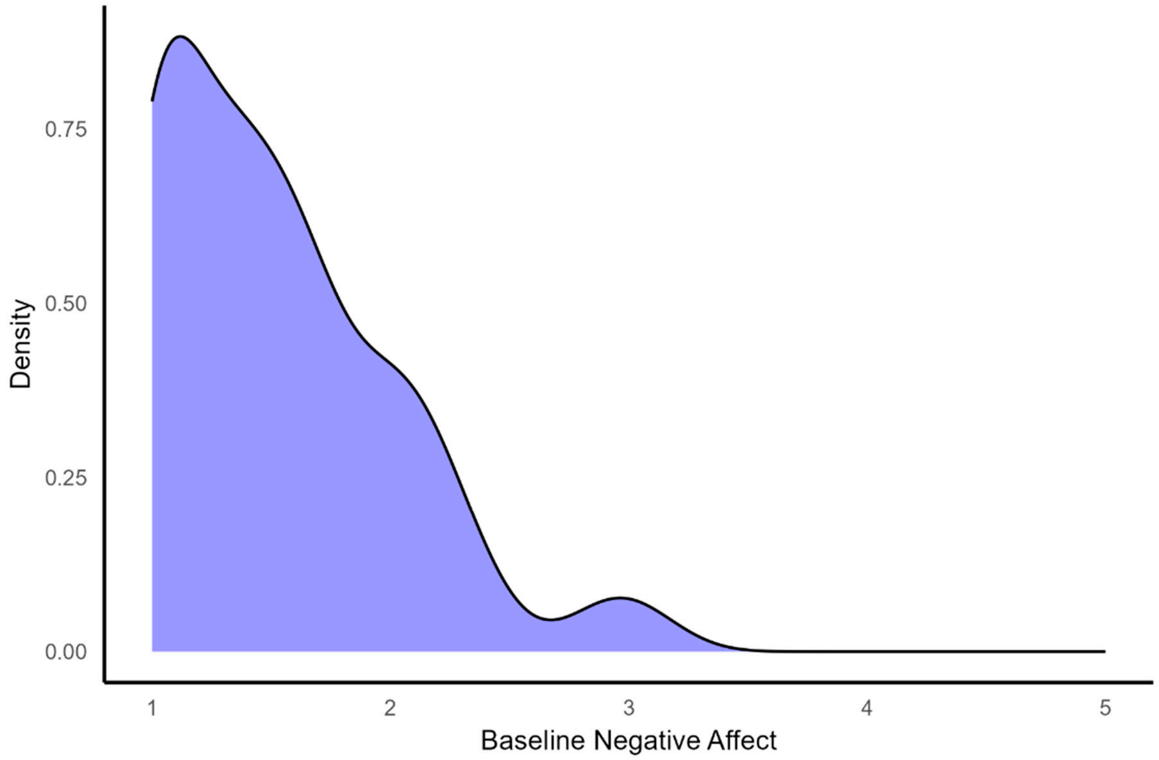

Figure S4. Density Plot of Negative Affect at Baseline (*N* = 156)

Section S1. Post Hoc Exploratory Results

To gain further insight into the observed null findings, additional factors were explored as potential moderators. The section below provides the detailed results of these analyses.

First, sex was examined. For positive affect, sex showed no significant main effect ($B = 0.24$, $SE = 0.13$, $p = 0.06$), nor did it moderate changes over time (Day × Sex; $B = -0.01$, $SE = 0.01$, $p = 0.40$) or group differences (Day × Group × Sex; $B = 0.006$, $SE = 0.02$, $p = 0.69$). For negative affect, sex showed no significant main effect ($B = 0.12$, $SE = 0.10$, $p = 0.26$), and did not moderate changes over time (Day × Sex; $B = -.002$, $SE = 0.01$, $p = 0.87$) or group differences (Day × Group × Sex; $B = 0.002$, $SE = 0.01$, $p = 0.91$).

Next, baseline positive and negative affect were examined in relation to their respective affect. Baseline positive affect showed a significant main effect ($B = 0.81$, $SE = 0.06$, $p < 0.001$), indicating that youth with a higher level of baseline positive affect reported a higher level of positive affect during the study. Baseline positive affect also significantly moderated change over time (Day × Baseline Positive Affect; $B = -0.03$, $SE = 0.04$, $p = 0.003$), such that youth with higher baseline positive affect showed less change. There was no significant moderation by group (Day × Group × Baseline Positive Affect; $B = 0.001$, $SE = 0.01$, $p = 0.94$).

For negative affect, baseline negative affect showed a main effect ($B = 0.78$, $SE = 0.017$, $p < 0.001$), indicating that youth with a higher level of negative affect at baseline reported higher daily negative affect across the study. Baseline negative affect also significantly moderated change over time (Day × Baseline Negative Affect; $B = -0.03$, $SE = 0.01$, $p = 0.03$), indicating that youth who started out with higher negative affect showed less change in negative affect across the study. There was no significant moderation by group (Day × Group × Baseline Negative Affect; $B = 0.003$, $SE = 0.02$, $p = 0.86$).

Finally, in the training group, task completion was explored as potential moderator. Task completion was defined as the number of days participants reported completing their chosen goal-directed behavior, with a maximum of 12 days. Half of the participants indicated task completion on seven or more days. For positive affect, task completion did not moderate changes in positive affect over time (Day × Task Completion; $B = 0.003$, $SE = 0.003$, $p = 0.30$). For negative affect, there was a main effect of task completion ($B = 0.05$, $SE = 0.02$, $p = 0.03$) but no significant interaction between time and task completion (Day × Task Completion; $B = -0.005$, $SE = 0.003$, $p = 0.050$). The small main effect should be interpreted with caution, given the relatively small number of participants (i.e., training group, $n = 72$) and the multiple post-hoc tests conducted.
